# Cycle Length Evaluation in Persistent Atrial Fibrillation Using Kernel Density Estimation to Identify Transient and Stable Rapid Atrial Activity

**DOI:** 10.1007/s13239-021-00568-1

**Published:** 2021-08-27

**Authors:** Szabolcs Z. Nagy, Patrick Kasi, Valtino X. Afonso, Nathaniel Bird, Brian Pederson, Ian E. Mann, Steven Kim, Nicholas W. F. Linton, David C. Lefroy, Zachary I. Whinnett, Fu Siong Ng, Michael Koa-Wing, Prapa Kanagaratnam, Nicholas S. Peters, Norman A. Qureshi, Phang Boon Lim

**Affiliations:** 1 Imperial College London, Hammersmith Hospital, 72 Du Cane Rd, London, W12 0HS United Kingdom; 2 Abbott Inc, One St Jude Medical Dr, St Paul, MN 55117 USA

**Keywords:** Biomedical signal processing, Cardiology, Intracardiac electrograms, Extra pulmonary vein drivers, Ablation

## Abstract

**Purpose:**

Left atrial (LA) rapid AF activity has been shown to co-localise with areas of successful atrial fibrillation termination by catheter ablation. We describe a technique that identifies rapid and regular activity.

**Methods:**

Eight-second AF electrograms were recorded from LA regions during ablation for psAF. Local activation was annotated manually on bipolar signals and where these were of poor quality, we inspected unipolar signals. Dominant cycle length (DCL) was calculated from annotation pairs representing a single activation interval, using a probability density function (PDF) with kernel density estimation. Cumulative annotation duration compared to total segment length defined electrogram quality. DCL results were compared to dominant frequency (DF) and averaging.

**Results:**

In total 507 8 s AF segments were analysed from 7 patients. Spearman’s correlation coefficient was 0.758 between independent annotators (*P* < 0.001), 0.837–0.94 between 8 s and ≥ 4 s segments (*P* < 0.001), 0.541 between DCL and DF (*P* < 0.001), and 0.79 between DCL and averaging (*P* < 0.001). Poorer segment organization gave greater errors between DCL and DF.

**Conclusion:**

DCL identifies rapid atrial activity that may represent psAF drivers. This study uses DCL as a tool to evaluate the dynamic, patient specific properties of psAF by identifying rapid and regular activity. If automated, this technique could rapidly identify areas for ablation in psAF.

## Introduction

Rapid cycle length activity (110–270 ms) from the pulmonary veins plays a role in the initiation of atrial fibrillation (AF).^12^ Electrical isolation of the pulmonary veins (PVI) can have success rates of 69–89% in paroxysmal AF.^24^,^4^ In persistent AF (psAF) however, success rates are lower, and show variation (21–74%).^2^ This discrepancy may be caused by the remodelling of left atrium (LA) in psAF, that creates complex driving mechanisms outside of the pulmonary veins. Linear ablation lesions or complex fractionated electrogram ablation have not shown incremental value when added to PVI.^34^

Atrial fibrillation cycle length (AFCL), measured as local activations determined from intracardiac electrograms in the atria, has been used to assess response to PVI, vagal stimulation, complex fractionated electrogram ablation, and interrogation of regional differences in AF with averaging over consecutive cycles.^13^,^16^,^20^,^25^

Non-pulmonary vein drivers of psAF may manifest as rapid AFCL, high frequency areas, that exhibit rotational activity, with gradients within the atrial tissue.^21^ Semi-automated techniques to identify dynamic drivers using simultaneous or sequential phase mapping,^22^ dominant frequency mapping,^1^ spatio-temporal dispersion assessment^29^ and wavefront propagation evaluation^14^ have shown promising results, but multi-centre reproducibility is yet to be proven. There has been promising research on using AFCL to locate areas with a low effective atrial refractory period that may contribute to psAF maintenance.^35^,^32^ The mechanisms underlying non-pulmonary vein AF drivers remain poorly understood.

Dominant frequency (DF) analysis using Fast Fourier Transform (FFT) uses local atrial signals to identify areas of rapid activity.^27^ DF results however can vary significantly if the processed signal has variable amplitudes or variable frequency.^23^,^28^ A comparison of AFCL with DF showed poor correlation.^8^.

Recent research has shown that areas where catheter ablation resulted in acute termination of psAF, demonstrated rapid, regular and organised activity.^18^ The currently accepted method of determining cycle length by simple averaging is difficult to apply to AF segments with variable electrogram quality secondary to intermittent contact with the atrial endocardium.

The following manuscript introduces a novel approach to measuring AFCL. Unlike simple averaging of manual annotations^13^,^16^,^20^,^25^ and DF analysis, our approach does not assume a normal distribution of local atrial activations or unchanging amplitude and cycle length. Our AFCL evaluation method, dominant cycle length (DCL), surmounts the issue of transient loss of electrograms that may skew averages and distort results. We use DCL to locate areas of rapid activation, that might serve as targets for extra-pulmonary vein driver ablation in psAF. We introduce metrics that describe the quality of electrograms, and consistency of AF within a time period.

## Methods

Seven patients (61 ± 11 years of age) undergoing first time ablation for symptomatic psAF (> 7 days) were included. All patients provided written informed consent for the export and analysis of their electroanatomical mapping data. Data were used from two studies with approval from the UK National Research Ethics Service (14/LO/1367; 17/LO/0524). The procedures were in accordance with the ethical standards of the Helsinki Declaration of 1975 as revised in 2000.

### Electrophysiology Procedure

We used EnSite Precision™ (Abbott Inc, St Paul, MN, USA), a non-fluoroscopic navigation system through which all electrodes in the heart are visualized within the electric field generated via six surface electrodes creating three orthogonal axes with the heart in their centre.^33^ LA electroanatomical data were collected using the AFocus II™ spiral mapping catheter with 20 poles and a 4 mm spacing with a 20 mm loop diameter (Abbott Inc, St Paul, MN, USA). Complete LA maps were created prior to PVI in non-cardiac triggered mode, that enables the user to collect up to 8 s of electrogram data for every bipole. Recording was automatically started in stable catheter positions (< 10 mm catheter movement). Eight-second electrograms were collected for all 19 bipoles of the AFocus II. Points within 7 mm of the geometry shell were included in the map and the minimum interpolation distance for map colour was set at 7mm.

### Annotation and Analysis of Electrograms

**Figure 1:**
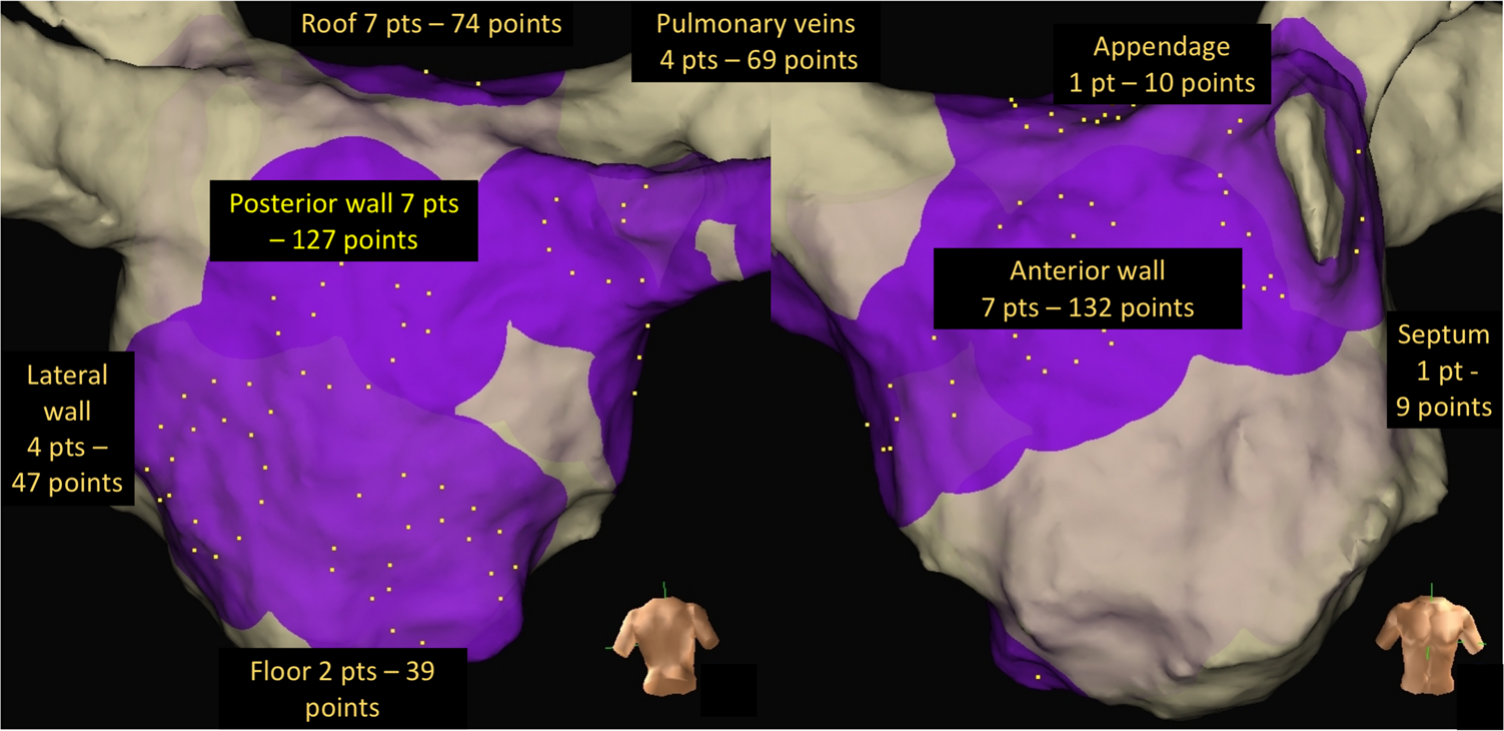
Electrogram samples were collected from all major regions of the left atrium to ensure that the algorithm was tested on electrograms with various characteristics.

**Figure 2:**
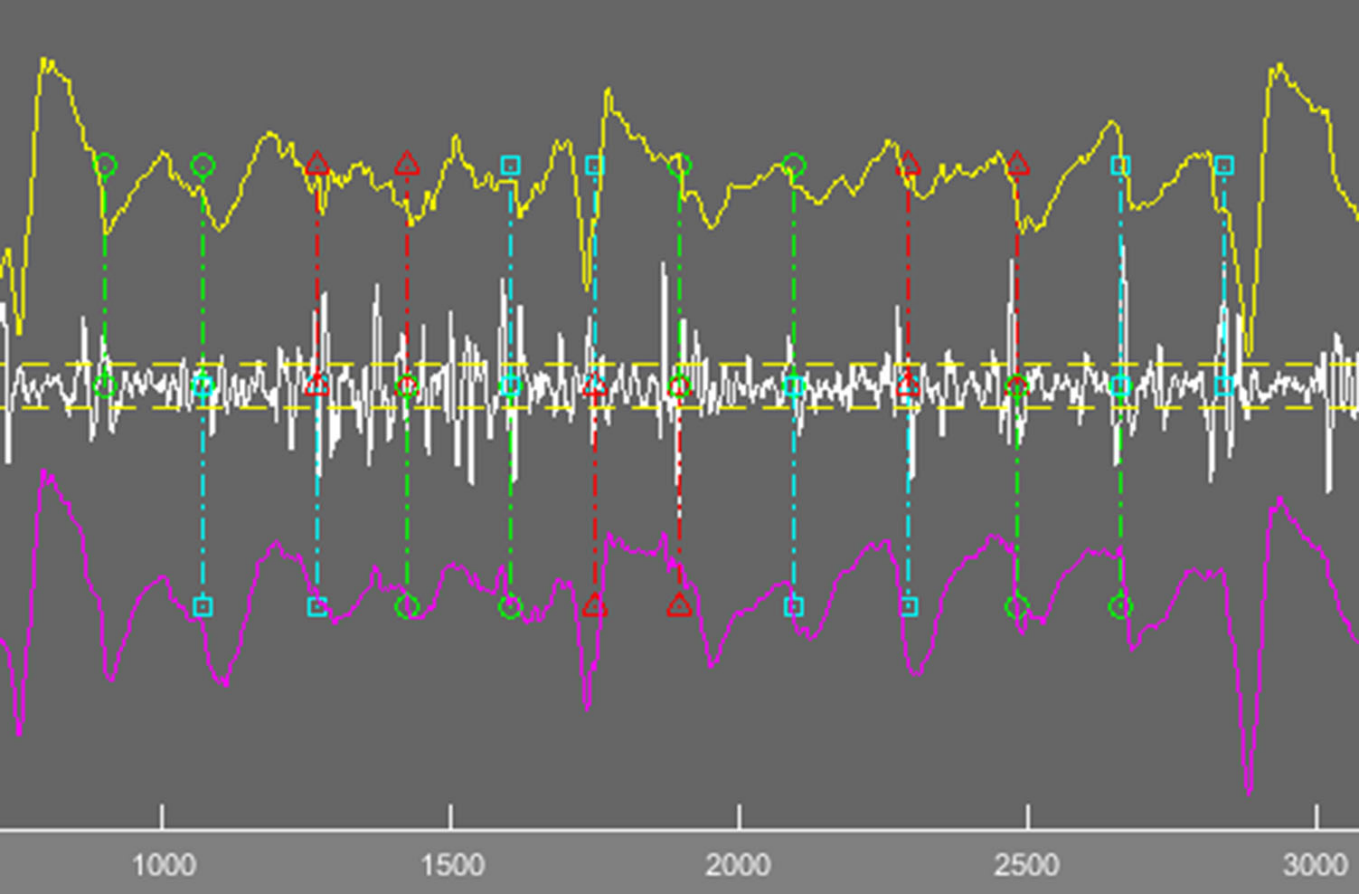
Use of unipolar electrogram in identifying local activations. In case of difficult to interpret bipolar electrograms, the corresponding unipolar electrograms can be loaded into the EnSite Electrogram Analysis Tool (EEAT) enabling identification of local activation based on maximum negative dV/dt. Local activations are assessed in pairs. From the left, the pair marked with green concludes at the same point that the next annotation pair marked in blue starts, thereby ensuring that all local activation pairs are taken individually.

Focused electroanatomical maps with up to 10 catheter locations (with 19 bipolar electrograms) were created retrospectively for every patient using the Ensite Precision™ Research Software, to allow assessment of various signal qualities from various locations (Fig. 1) . Data were imported into the EnSite Electrogram Analysis Tool (EEAT) for electrogram annotation and analysis based on MATLAB® (Mathworks Inc, Natick, MA, USA), developed within the research group. Electrograms for analysis were chosen randomly, ensuring an even spread of samples within a patient. Annotation of local atrial activations to determine DCL was done manually, by visual inspection of bipolar electrograms. Annotated landmarks corresponded to the largest peak. In situations where the bipolar signals we difficult to interpret, unipolar signals were inspected, and the steepest dV/dt slope was used as the annotation landmark. Each adjacent annotation pair in a signal constituted one measurement (ms). Uninterpretable segments where the signal to noise ratio did not permit a clear distinction of local activations, were considered electrical noise (Fig. 2). Electrograms with ≤ 5 CL pairs were excluded.

To assess inter-observer variability, a subset of electrograms were annotated by two annotators, who were blinded to each other’s annotations. Operator 1 assessed all 507 electrograms and operator 2 annotated 293 (58%).

Local activations with low voltage (< 0.05 mV) were excluded from all analyses. As histogram plots of local activations from each 8 s segment showed a broad range of unimodal to multimodal distributions, mean or median AFCL was deemed inadequate to describe activity within a segment. This suggested that kernel density estimation (KDE) was appropriate for DCL analysis.

#### Kernel Density Estimation

KDE is a non-parametric method to estimate the probability density function (PDF) of a continuous random variable. The KDE is

$$\hat{f}\left( x \right) = \frac{1}{n}\mathop \sum \limits_{i = 1}^{n} \frac{1}{h}K\left( {\frac{{x - x_{i} }}{h}} \right)$$

where *K* is the kernel function, and *h* is the bandwidth of the kernel.

**Figure 3:**
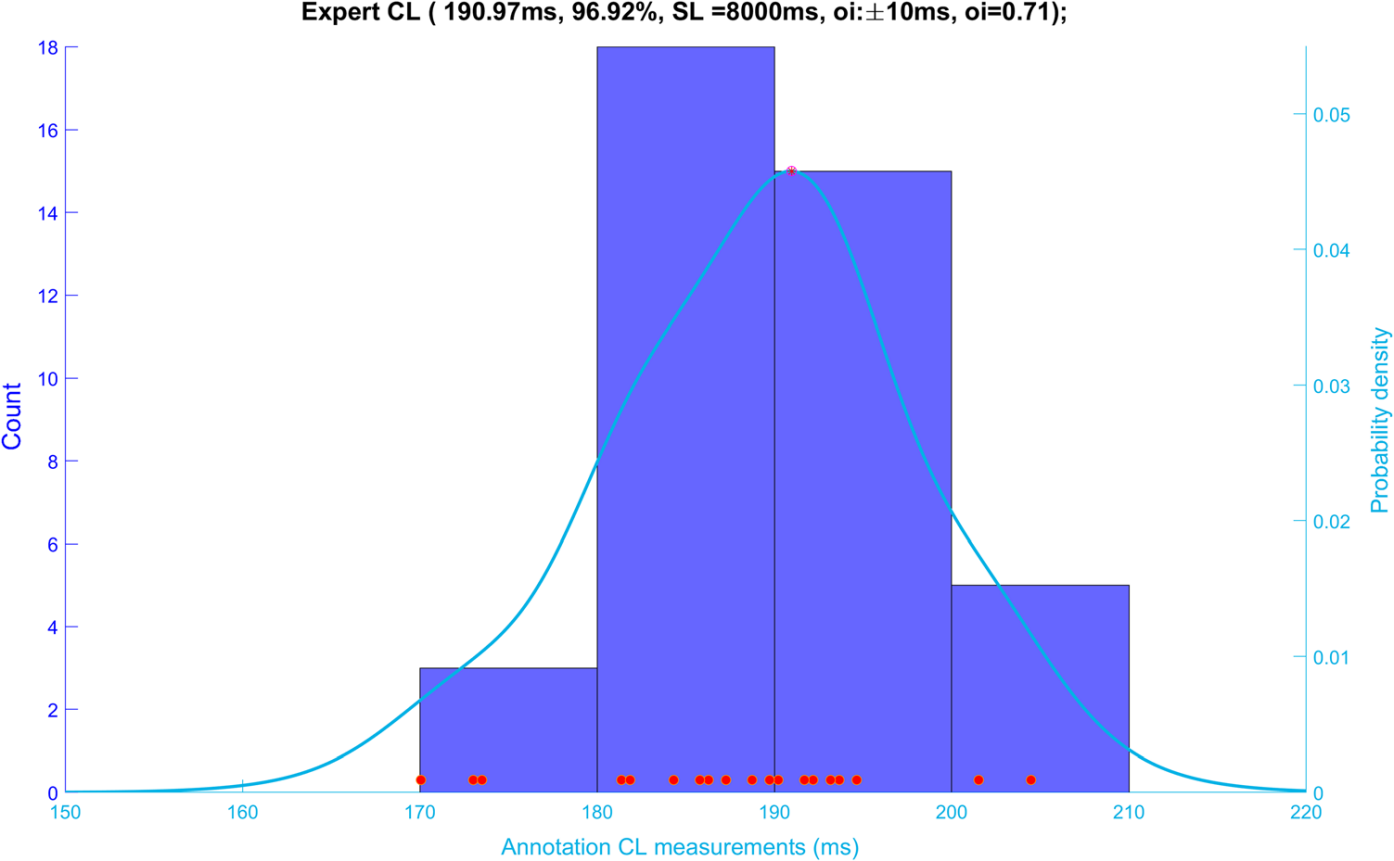
Kernel density estimation compared to histogram based analysis. *CL* AF cycle length from manual annotations, *SL* AF segment length, *OI* dominant cycle length organisational index, *n* local activation pair samples within the peak, *span* summative time of all local activation pairs within the peak.

KDE creates a kernel function at each CL measurement, and takes the sum of all kernels. Kernel and bandwidth in the above formula are at the operator’s discretion. We chose the computationally simple Gaussian kernel. The quality of the KDE is based on the width of the kernel function (bandwidth). When the bandwidth is wide, the density estimate is oversmoothed. When the bandwidth is too small, the KDE has several spikes, making interpretation difficult. Based on experimentation, we chose a bandwidth of 5 ms that allowed for distinction of PDF modes corresponding to local activation clusters that were at least 20 ms apart (Fig. 3).

#### Determining Dominant Cycle Length

**Figure 4:**
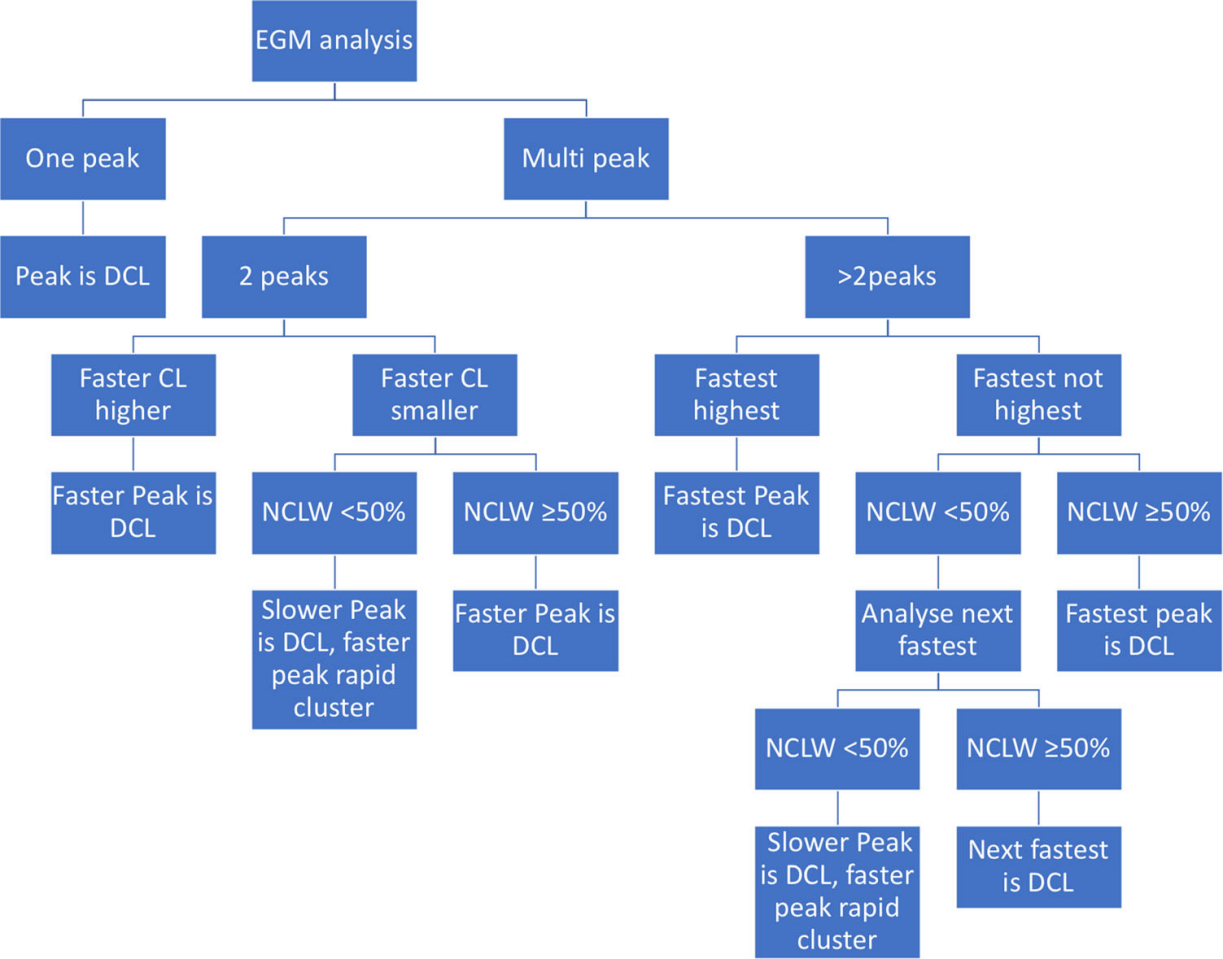
Flowchart representing the algorithm used to define dominant cycle length and rapid cluster cycle length within each 8 s atrial fibrillation segment. DCL: dominant cycle length; CL: cycle length; NCLW: number of cycle length data in ± 5 ms window.

To describe the representative AFCL for a segment, we introduced the term DCL. DCL for each 8 s segment was computed form the PDF estimates as described below. Only valid DCL segments were used for analysis (DCL: 80–250 ms). We rejected all 8s AF segments whose DCL result fell out of the 80–250 ms range. Some PDF estimates possessed a single peak, others were multimodal. A peak was further analysed, if within a ± 5 ms window, there were ≥ 5 CLs measured. We considered 5 local activation pairs to ensure that the identified phenomenon is a representative CL rather than noise. We came to the conclusion that 5 local activation pairs cover approximately 1s, if AF is rapid. This would be an approximate limit that could be identified by the human eye during a procedure, and it would also filter very short bursts that may not be manifestations of drivers. DCL was determined based on the number of annotation pairs in each peak window on the PDF estimate plot. In case of unimodal distributions, the single peak was chosen as DCL. For multimodal distributions, after identification of the largest PDF peak, all PDF peaks corresponding to faster CL values (compared to the CL of the largest PDF peak) were assessed (faster peaks). If any of the faster peaks was found to be ≥ 50% in size compared to the largest peak, this peak was chosen as DCL. In addition, if any of the faster peaks had ≥ 5 annotations pairs but was < 50% in size compared to the DCL peak, it was considered as a “rapid cluster” (Fig. 4). The rationale to focus the algorithm on more rapid activity within a segment arose from review of all segments and data published on the relevance of rapid AFCL in AF termination. On manual review, the 8s segments that contained rapid activity often contained rapid subsegments with a background of regular activity, at a longer cycle length. We hypothesised that the slower background activity is less likely to be the active driver and more likely represent passive activation. Our hypothesis was based on previously published data showing that slower AFCL overall predicts ablation success,^7^ termination of AF during ablation was associated with ablation at areas of rapid AFCL^18^ and drivers of AF co-locate with rapid areas.^15^

#### Segment Quality

Electrogram quality was represented by the proportion of segment length with valid local activation annotations in relation to complete segment length. Electrogram complexity was initially evaluated by the number of CL peaks with ≥ 5 annotation pairs within the peak window. The DCL organisation index (DCL-OI) was developed in order gain quantifiable information on electrogram complexity. DCL-OI was calculated by the ratio of the area defined by a ±10 ms window around the DCL peak, divided by area under the entire PDF. Similarly to the consistency metric introduced by Jadidi et al.,^18^ DCL-OI provides information on the scatter of local activation intervals from a value that describes the entire AF segment, with the additional dimension of DCL.

Highly organised segments were defined as those with a DCL-OI greater than the mean DCL-OI + 1 SD for a patient.^19^

#### Comparison with Dominant Frequency and Simple Averaging

Dominant frequency by FFT was determined for all intracardiac 8 s segments with concomitant surface ECGs (*N* = 500). Following QRS suppression guided by the surface ECG, the signal was band pass filtered (40–250 Hz), rectified and passed through a low pass filter. The power spectral density was estimated using Welch’s periodogram 27. Signals were sampled at 2034.5 Hz. We analysed 8-second electrogram epochs, and the frequency resolution is 0.125. The frequency in Hz of the highest peak of the power spectral density was converted to ms by inversion (DF derived CL). We calculated DF organisational index (DF-OI) as the ratio of the power band +/- 0.75 around the DF (area) to the power (area) of the interval 3–15 Hz band as described by Sanders et al.^26^ Valid results (80–250 ms) and measurement differences were compared with DCL results.

To compare DCL to “simple averaging”, the cumulative annotation length of annotations in ms was divided by the number of annotations-1 and plotted against DCL.

### Statistical Analyses

Continuous data were described as either mean value ± standard deviation or median and interquartile range. The proportion of valid results between DF and DCL were compared using the related-samples McNemar change test. DF versus DCL and inter-annotator variability were compared using Spearman’s and intra-class correlation and Bland Altman agreement. The Mann-Whitney U Test was used to assess differences in DCL-OI of large and small measurement error segments. Simple averaging and DCL were compared using Spearman’s correlation. Statistical significance was defined as *P* < 0.05.

## Results

**Figure 5:**
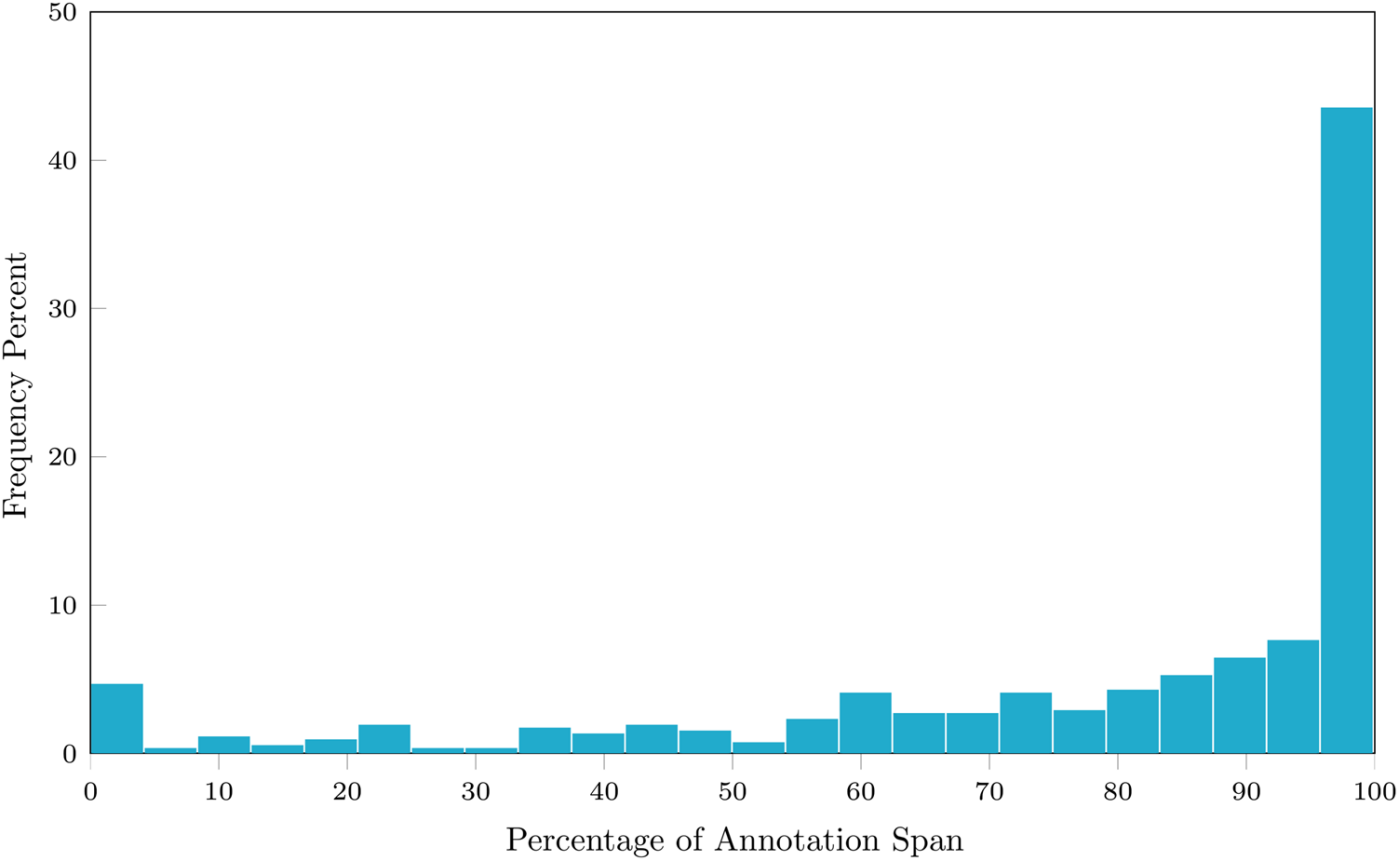
Frequency percentage of all valid segments by proportion of segment annotated. The majority of segments had annotations that covered ≥ 50% (4 s) of the segment.

Data were analysed from 7 patients (Age 61 ± 11, 3 female, AF duration: 23.1 ± 12.1 months, CHADSVASC 1.6 ± 1.4). Baseline characteristics are in Table 1 and Table 2. Of 507 8 s AF segments from various LA locations 450 valid segments were analysed (88.7%;). Mean DCL was 178 ± 28.6 ms (range 93–250 ms). Rapid cluster cycle length was found in 250 segments (55.6%; mean CL 159.6 ± 27.8; [range 78–231 ms]). The mean difference between DCL and rapid cluster CL was 24.4 ± 11.5 ms. Median annotation percentage was 95% (IQR: 21.9). 265 segments (58.9%) had ≥ 90%, and 419 segments (93.1%) had ≥ 50% valid annotations (Fig. 5).

**Table 1:** Baseline characteristics of patients included in the study.

Age (years)	61 ± 11
Male	4 (57.1)
Diabetes Mellitus	1 (14.3)
Hypertension	2 (28.6)
TIA/CVA	0 (0.0)
Ischaemic heart disease	1 (14.2)
Cardiac surgery	0 (0.0)
Left ventricular EF > 55%	6 (85.7)
LA size (diameter, according to British Society of Echocardiography Guidelines)	
Normal (30–40 mm)	3 (42.8)
Mild (4.1–4.6)	2 (28.6)
Moderate (4.7–5.2)	2 (28.6)
Severe (15.3)	0 (0.0)
AF duration(months)	23.1 ± 12.1
Current antiarrhythmic	
Beta-blockers	5 (71.4)
Amiodarone	2 (28.6)
Current anticoagulant	
Warfarin	4 (57.1)
Direct oral anticoagulants	3 (42.9)
Number of electrograms analyzed	70 ± 24
Mean valid DCL (ms)	180 ± 27
CHA_2_-DS_2_-VAS_c_ score	
3	3 (42.9)
1	2 (28.6)
0	2 (28.6)

**Table 2:** Number of annotated electrograms per patient included in the study, with DCL characteristics DCL: Dominant cycle length; STDEV: Standard deviation.

Subject ID	Annotated electrograms	Computed DCL		
	Number of annotated electrograms	Number of electrograms used for analysis	DCL mean	DCL STDEV
1	100	97	183	10.8
2	79	72	158	19.5
3	38	23	163	32.5
4	47	41	170	22.6
5	65	64	186	11.1
6	78	61	130	14.3
7	100	92	193	43.5
Total		507		450

### Inter-observer Variability

**Figure 6:**
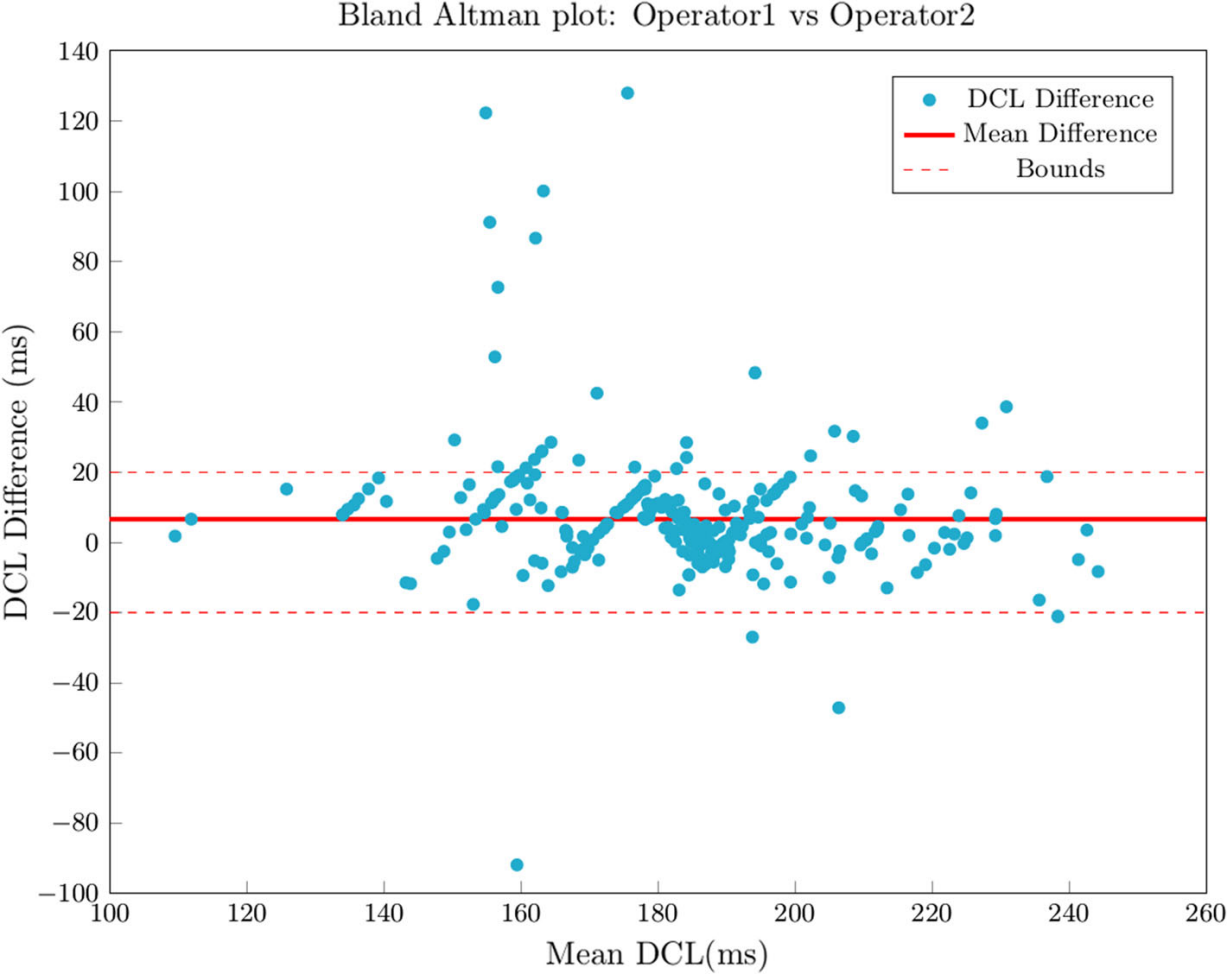
Bland Altman plots describing measurement differences between Operator 1 vs Operator 2. Red line represents mean difference.

All electrograms were annotated by Operator 1. Operator 2 annotated 293 electrograms, yielding 289 valid results. All analyses were performed in segments with a valid DCL by both Operator 1 and Operator 2 (287; 99.3%). Correlation between operators was strong (Spearman’s rho: 0.758; *P* < 0.001) and intraclass correlation indicating interrater reliability was moderate (ICC: 0.696; *P* < 0.001). Median error was 6.4 ms (IQR:10), and 257 segments (89.5%) had a measurement error ≤20 ms (Fig. 6).

### Consistency and Variability of DCL

**Figure 7:**
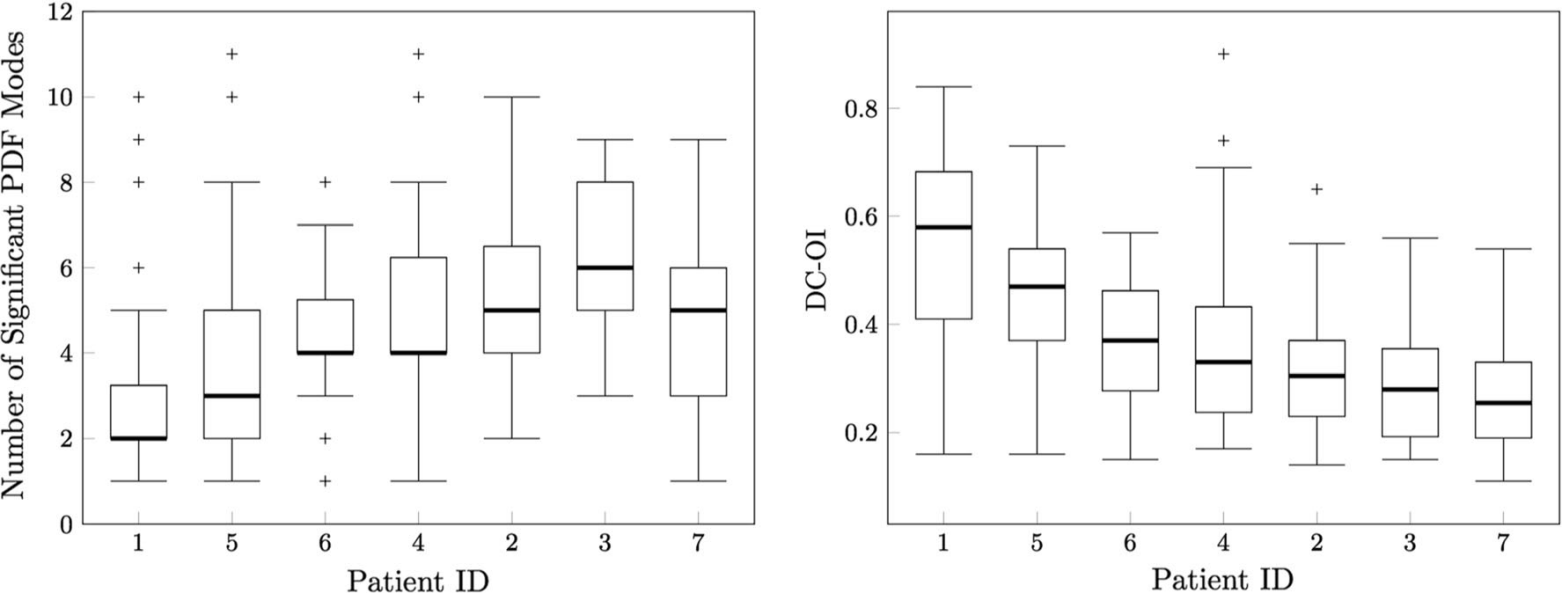
Dominant cycle length organisational index (DCL-OI) and Peak number by patient. Patient 1 had the highest DCL-OI and a low number of segments with peak count > 2.

**Figure 8:**
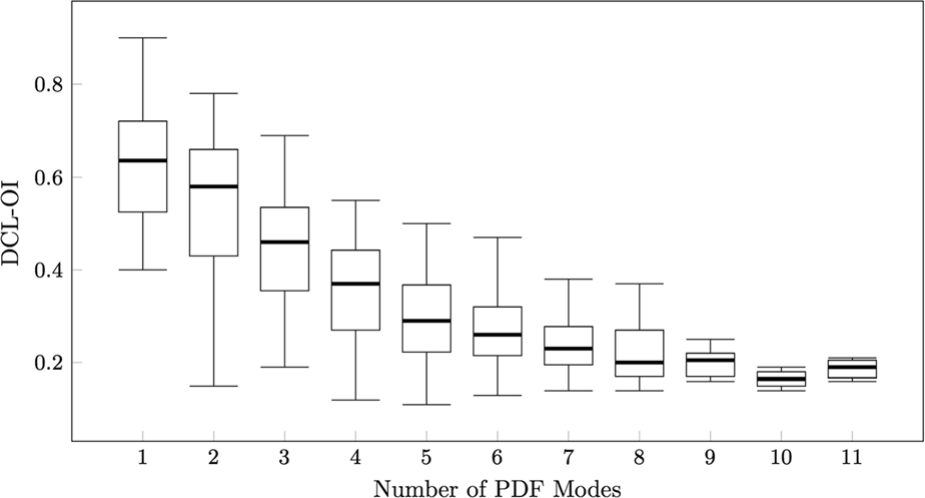
The relationship between dominant cycle length organisational index (DCL-OI) and peak number shows a tendency for higher DCL-OI with fewer peaks.

**Figure 9:**
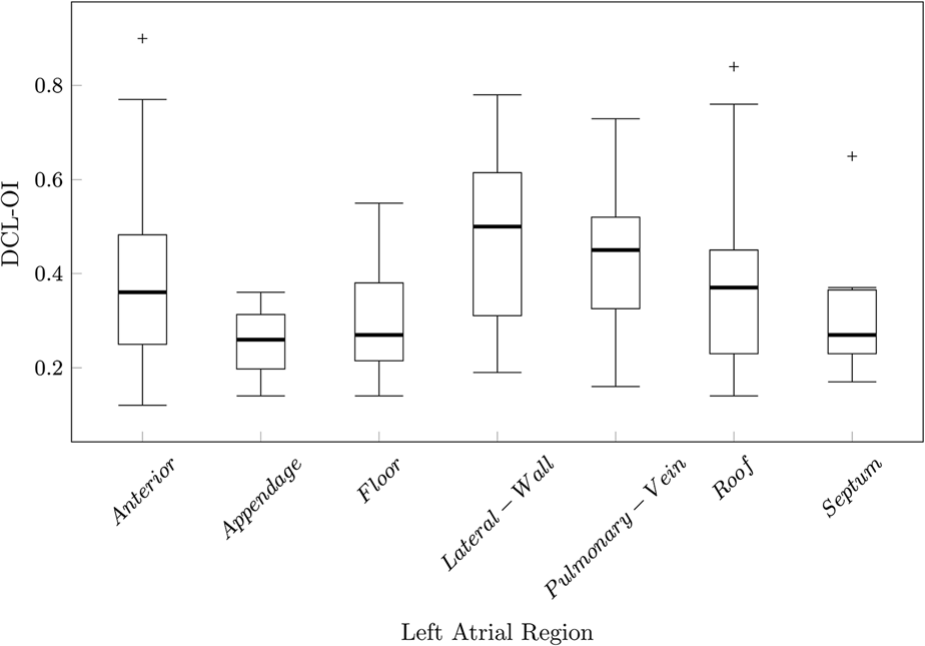
Regional distribution of Dominant cycle length organisational index (DCL-OI) .

**Figure 10:**
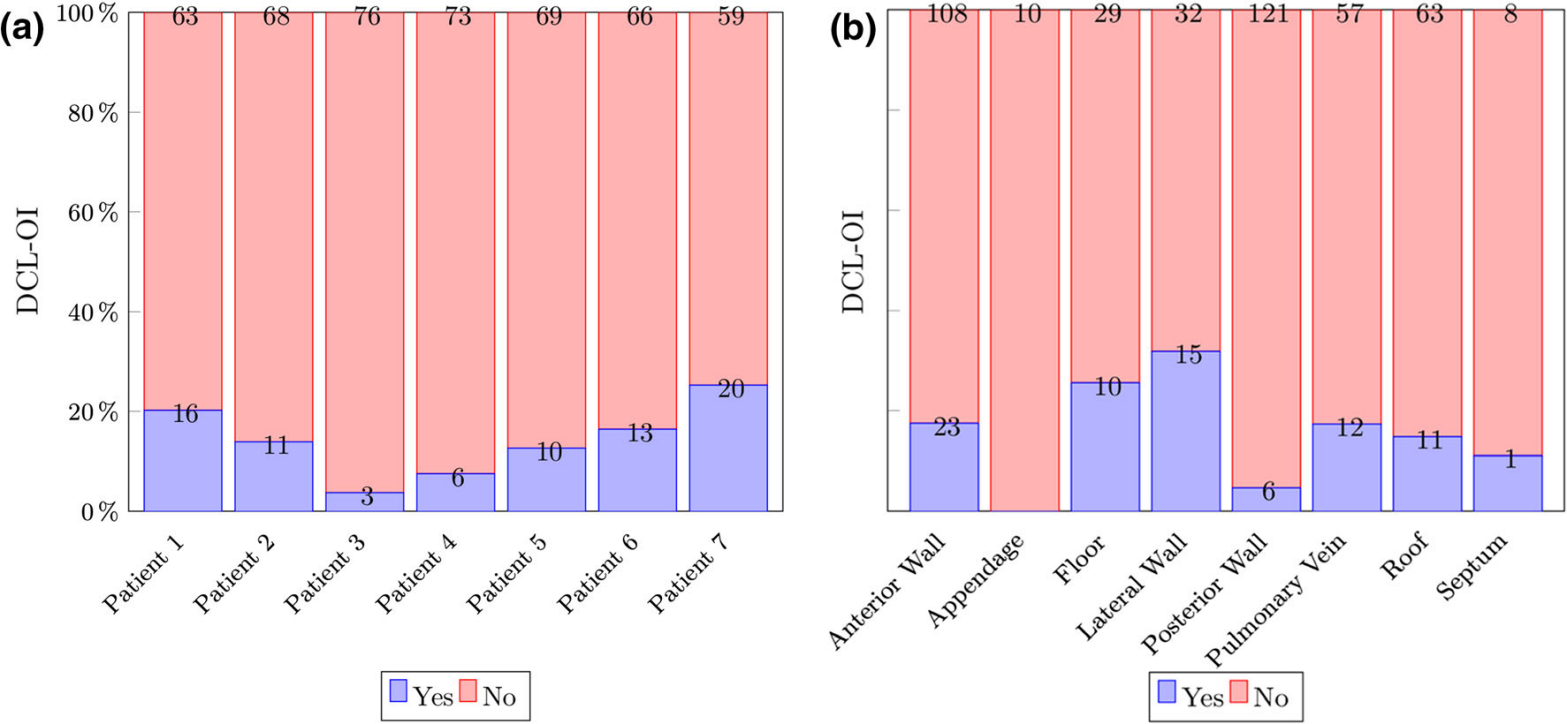
Percentage of highly organised areas according to Dominant cycle length organisational index (DCL-OI), by patient (A) and by region. (B). Highly organized areas appear in blue.

Median DCL-OI for all patients was 0.36 (IQR:0.24) and median peak number was 4 (IQR:3). There was a strong negative correlation between the peak number and DCL-OI (Spearman’s rho: − 0.755; *P* < 0.001). DCL-OI and mean peak number by patient can be found in Fig. 7. A negative trend was observed between DCL-OI and peak number (Fig. 8). DCL-OI by region can be seen on Fig. 9. The mean percentage of highly organised segments by patient and by region was 17.2 ± 3.3% (range 13–21.7%) and 18.5 ± 13.8% (range 0–45.7%) respectively, with the most highly organised segments on the lateral wall, and the fewest in the appendage (Fig. 10).

### Comparison with DF

**Figure 11:**
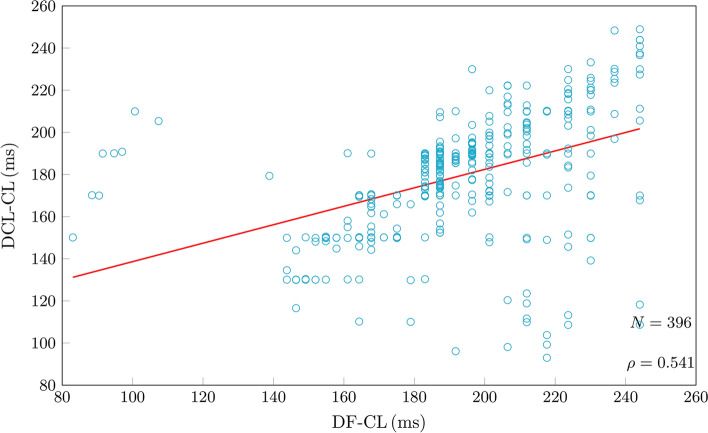
Comparison of manual dominant cycle length (DCL) results and cycle length results based on dominant frequency (DF) analysis. There was moderate correlation and intraclass correlation was poor.

**Figure 12:**
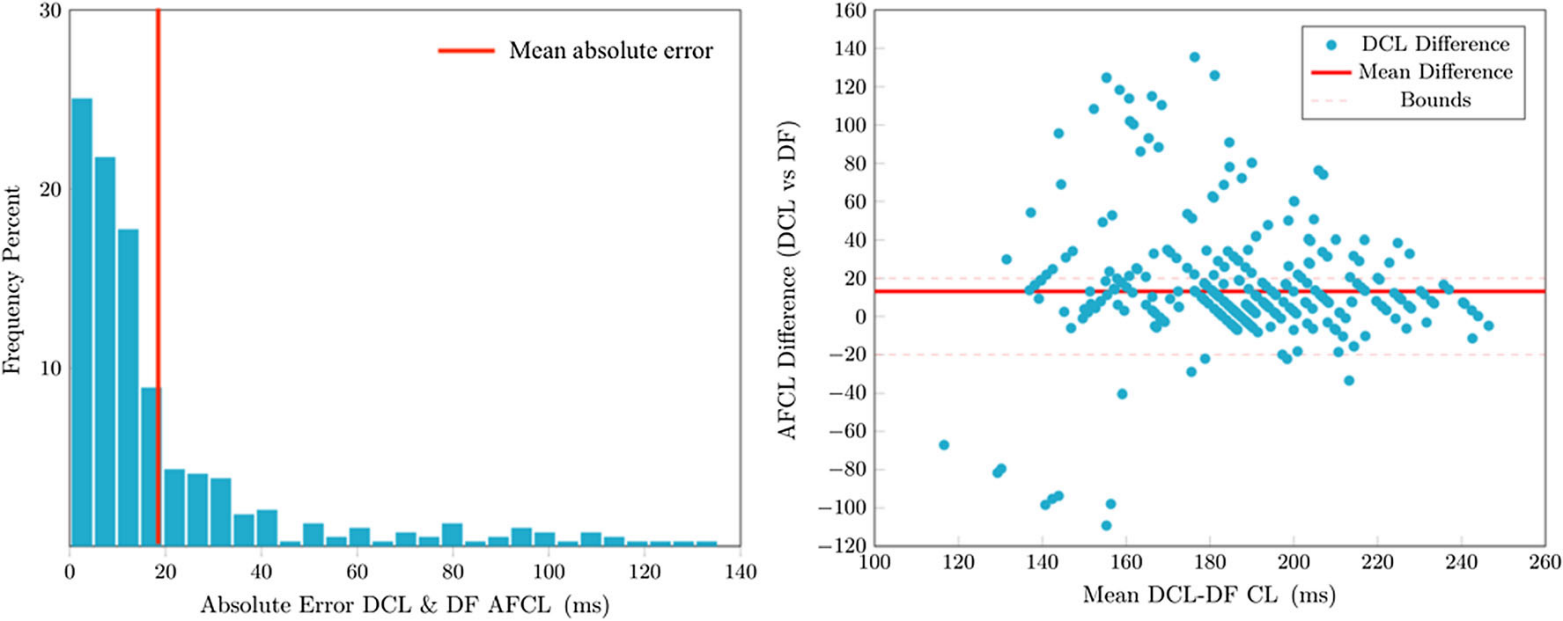
Absolute measurement errors of dominant frequency-based (DF) cycle length compared with manual dominant cycle length (DCL) (left). Bland Altman plot of differences between DF based CL and manual DCL. Red line represents mean difference (right).

**Figure 13:**
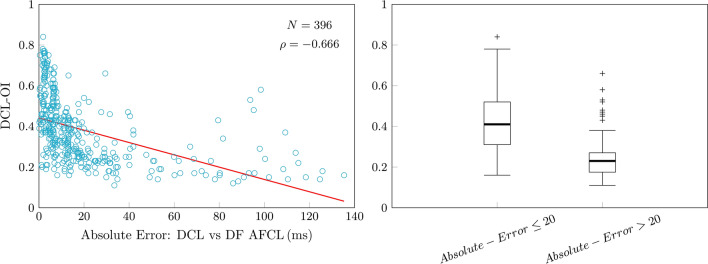
Dominant cycle length organisational index (DCL-OI) plotted against absolute measurement errors between dominant frequency-based (DF) results and dominant cyle length (DCL) results (left). Box plot of DCL-OI of segments that had > 20 ms measurement difference compared with those that had  < 20 ms measurement disagreement.

DF analysis returned valid values in 415 (81.9%) cases, compared to 450 (88.7%) with DCL (*P* < 0.001). The number of false positive results compared to DCL was 19 (4.2%), 54 (12%) were false negative. Correlation between DCL and DF derived CL was moderate (rho: 0.541; *P* < 0.001) and intraclass correlation was poor (0.442; *P* < 0.001;Fig. 11). Correlation between DCL-OI and DF-OI was weak (rho: 0.28; *P* < 0.001) and intraclass correlation was poor (0.21; Appendix). Median absolute error for valid measurements was 11.2 ms (IQR:16), mean absolute error was 19.5 ms (SD ± 25) and 292 (73.7%) measurements had ≤20 ms error (Fig. 12). DCL-OI had a strong negative correlation with measurement differences between DF derived CL compared to DCL (Spearman’s rho: − 0.666; *P* < 0.001) and DCL-OI was significantly greater for electrograms with measurement differences ≤20 ms (0.41 [IQR 0.10] vs 0.23 [IQR 0.1]; *P* < 0.001; Fig. 13 ).

### Comparison with Simple Averaging

**Figure 14:**
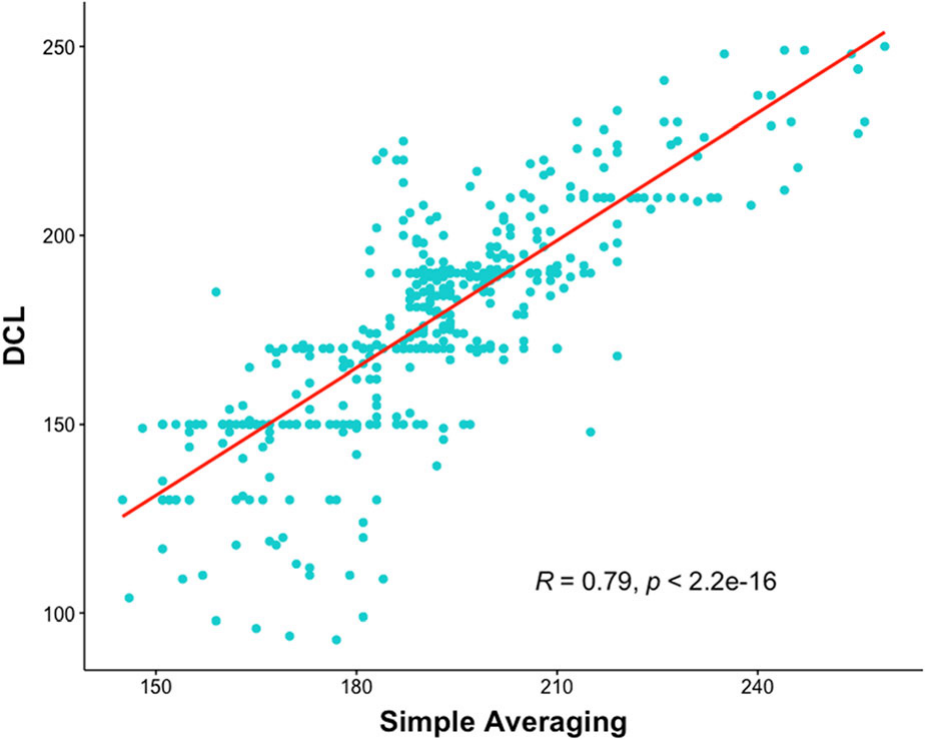
Correlation of results from the dominant cycle length (DCL) algorithm compared with simple averaging. Linear regression line in red, R = Spearman’s rho.

We compared all valid DCL results with simple averaging. For all AF segments (*N* = 450), correlation was very strong (rho: 0.79; *P* < 0.001; Fig. 14).

## Discussion

### Main Findings

To the best of our knowledge, this is the first study that uses metrics derived from direct local activation measurement beyond simple histogram plots to describe extra pulmonary drivers and rapid activity in psAF. We calculated DCL, using an objective, step-by-step method. DCL discards segments with a significant proportion of electrical noise. Rapid cluster cycle length was conceived to identify transient rapid activity within an AF segment. Variability within an AF segment was assessed by the number of cycle length components (peaks) within a segment and by the relationship of the dominant cycle length to the entire recorded segment (DCL-OI). There was moderate correlation of DF to DCL, with larger measurement errors seen for less organised AF segments.

### Signal Quality and Reproducibility

Identification of non-pulmonary vein drivers of psAF has proven challenging. Some approaches use complex signal processing and a global view of the LA to identify drivers^22^,^14^,^5^ and some use sequentially collected local electrograms in combination with non-automated activation pattern recognition.^29^,^17^.

To date, only Focal Impulse and Rotor Modulation (FIRM) has had an attempt at multi-centre implementation, with variable results.^9^,^30^ This may in part be due to the complex interpretation of these maps, and in part be due to the subjective identification of rotational drivers and activation patterns thought to play a role in sustaining psAF.^22^,^29^,^14^,^17^

Although DF based ablation does not require subjective assessment of signals, visual assessment electrogram recordings is needed prior to DF signal processing to avoid false detection of high activation rates.^23^,^6^

If electrograms are reliably and consistently annotated, DCL provides access to unprocessed activation information and gives information without the need for extensive signal processing.

There is no strong evidence about the spatial and temporal stability of non-pulmonary vein drivers in psAF. Some suggest the meandering of drivers in the LA with few rotations in each location, showing repetitive recurrences in the same area, although the number of rotations that constitute a driver varies widely by research group.^14^,^10^,^3^ Others report excellent regional stability of rotational activity^31^ and the predilection of rotational drivers to certain low voltage areas.^14^ An *ex-vivo* study performed with simultaneous endo-epicardial optical mapping showed intramural microanatomic re-entry circuits during AF, that might be responsible for AF maintenance. However, owing to the 3-dimensional nature of these circuits, endocardial mapping might only uncover rapid activity that is temporally unstable but spatially stable, recurring in the same location.^11^ Previous work used average CL determined by manual or automated counts over arbitrary time periods as a metric of ablation success,^13^,^8^ disregarding the variability of AF.

DCL allows for identification of endocardial rapid atrial activity, that may highlight atrial fibrillation drivers. Complementing DCL, rapid cluster cycle length allows for the identification of more transient rapid activity within complex signals. Thus, rapid cluster cycle length may possibly represent drivers that fleetingly manifest on the endocardial surface but play a role in perpetuating psAF. Rapid cluster CL was found in more than half of the electrograms with valid DCL results. This supports the clinical observation of AF variability even during very short, 8 s segments.

The DCL algorithm focuses on highly regular activity at shorter segment lengths. This carries within it, the potential to highlight highly regular and rapid electrogram segments that have the hallmarks of an AF driver even with short duration mapping.

For data collection and automated segment analysis, ensuring adequate quality electrogram segments is crucially important.^6^ Segment quality was described by the simple measure of the cumulative duration of annotations divided by the total segment length. This metric can be used as a rapid assessment of the quality of collected electrogram segments and the reliability of the DCL results, as it describes what proportion of the recorded segment was used to determine DCL calculation. In the clinical setting, a low annotation percentage will highlight areas with rapid activity that would benefit from further interrogation to improve map accuracy.

Although different techniques have shown rapid rotational activity to persist for variable lengths of time,^14^,^10^,^3^ the role of AF variability is uncertain. The simple measure of local activation peak numbers within a segment provides an insight into signal complexity. DCL-OI gives more detailed information about the relevance of the DCL value and also the consistency of the segment. It uses, as its foundation, an established method of ascertaining consistency with a view of locating AF drivers.^19^ DCL-OI, when calculated in a clinical setting for a global 3D psAF map, may provide information about the relevance of the DCL result, taking into account the area under the curve of the DCL peak in relation to the power of the annotated segment. A higher DCL-OI might also indicate an area of higher consistency that is more likely to harbour AF drivers and thus be a more favourable target for ablation.

Our results indicate that the rapidity and consistency level of psAF differs between individuals and ablation strategies based on these metrics will need to be tailored to the patients’ psAF phenotype. This discrepancy between the regional and inter-patient variability of highly organised segments may be because the cut offs used to determine highly organised electrograms were specific to each patient, based on the distribution of patient-specific DCL-OI. This data appears to indicate that there are significant differences in regional consistency of psAF.

### Comparison with Dominant Frequency and Averaging

A significant body of research has dealt with the usage of dominant frequency to identify drivers of atrial fibrillation, despite evidence that DF has unpredictable performance with irregular electrograms with variable voltage^23^ and correlates poorly with CL measurements.^8^,^6^ Comparison with DCL shows that DF performs poorly returning measurement differences of > 20 ms in case of electrograms with lower consistency. Furthermore, while DF requires careful processing of signals prior to analysis to remove QRS, the DCL algorithm is less influenced by the outstanding, far slower, far-field QRS signals.

Simple averaging has been used as the gold standard methods in all previous publications measuring cycle length,^13^,^16^,^20^,^25^,^18^ it has significant limitations for high scale usage due to the manual annotation and pre-selection of electrograms. We have shown that our method is similarly capable of returning a cycle length as simple averaging, with the significant difference of the potential for automation, and the potential for determining DCL without pre-selection of electrograms based on quality.

### Limitations

We recognise the limitations of our study. Our low patient numbers, and resultant low electrogram segment numbers may affect the reproducibility of measurements. We collected data from 7 patients only and although signals were annotated from several different locations in the LA, complete LA maps were not analysed and segment length was limited to 8 s due to the time consuming nature of manual annotation. We recognize manual annotation as a significant limitation to the automation of this algorithm and realise that manual annotation introduces a measure of subjectivity. We are making efforts to fully automate the entire process. Due to the anatomy of the LA, some areas were more difficult to reach, therefore there was significant difference between patients, regarding the number of segments collected from certain regions. As with any study using contact mapping, intermittent contact with the LA endocardium may lead to intermittent signals. The DCL algorithm was developed to take into account intermittent signals and still return a relevant value. Rapid and regular DCL gives a good indication of possible driver regions but wavefront direction was not assessed. Although there are certain studies that indicated the role of transient rapid activity in the perpetuation of psAF, rapid activity has not been directly shown to play a role in driving AF. By using DCL from 80 to 250 ms, we excluded heavily fractionated signals, that may, in theory, represent drivers. By the exclusion of these excessively short DCLs we focused on distinct sharp deflections that may be identified as DCL with more certainty. We also acknowledge that by choosing rapid peaks ≥ 50% in size compared to the largest peak we introduced a bias towards shorter, more rapid bursts of activity.

## Conclusions

This work provides an introduction of DCL, an algorithm to describe local activation rates in psAF, with a view to identifying non-pulmonary vein drivers. The algorithm calculates DCL directly from detections of bipolar activations, and with the addition of rapid cluster cycle length, has the potential to identify transient, as well as temporally stable driver regions. Uniquely, the annotation length to segment length relationship is able to inform the operator about the quality of electrograms used to determine the DCL value, thereby identifying areas that would benefit from additional interrogation. DCL-OI highlights highly organised activity where the DCL cycle length takes up a significant proportion of the electrogram, and might play a significant role in sustaining psAF, directing the operator to potentially relevant areas for ablation. An automated technique of reliable and consistent annotation and automation of our algorithm is needed for widespread implementation of DCL mapping.

## Data Availability

Anonymised data and material used for this study are available for collaboration on request to the first author of this work. Code for the EEAT software in MATLAB is property of Abbott, Inc and is not available for sharing.

## Appendix

Comparison of manual dominant cycle length (DCL) results and atrial fibrillation cycle length (AFCL) results based on dominant frequency (DF) analysis. There was moderate correlation and intraclass correlation was poor.
